# Asiatic Acid Disrupts the Biofilm Virulence of *Streptococcus mutans* by Transcriptional Reprogramming of Quorum Sensing System

**DOI:** 10.3390/ijms26199510

**Published:** 2025-09-29

**Authors:** Qingying Shi, Fengzhu Li, Yingying Peng, Qiannan Sun, Hong Zhao, Fuping Lu, Huabing Zhao

**Affiliations:** 1 Key Laboratory of Industrial Fermentation Microbiology, Ministry of Education, College of Biotechnology, Tianjin University of Science and Technology, 9 TEDA 13th Street, Tianjin 300457, China; shiqingying@tust.edu.cn (Q.S.);; 2 State Key Laboratory of Food Science and Resources, Nanchang University, No. 235 Nanjing East Road, Nanchang 330031, China; 3 Tianjin Customs Animal, Plant, and Food Testing Center, No. 51, Second Avenue, Tianjin Economic-Technological Development Area (TEDA), Binhai New Area, Tianjin 300457, China

**Keywords:** *Streptococcus mutans*, asiatic acid, biofilm, dental caries, transcriptomics

## Abstract

Dental caries, a prevalent biofilm-mediated chronic disease, causes enamel demineralization, pulp infection, and systemic complications. Dental plaque biofilm is the initiating factor for the occurrence and development of caries. *Streptococcus mutans* is an opportunistic pathogen linked to the structure and ecology of dental plaque biofilms. The molecular mechanism of *S. mutans* during biofilm ontogeny in driving cariogenesis has been extensively elucidated. Here, we observed that asiatic acid is a potent biofilm disruptor that selectively dismantles *S. mutans* biofilm architectures, prompting us to investigate its mechanism. The minimum biofilm inhibition concentration (MBIC) of asiatic acid on *S. mutans* was 62.5 μM, but the MBIC level did not substantially impede planktonic growth. Using the static active-attachment model, it was demonstrated that asiatic acid significantly reduced biofilm biomass (*p* < 0.001) and extracellular polysaccharides (EPS) content (*p* < 0.001), while concurrently diminishing acid production (*p* = 0.017) and metabolic activity (*p* = 0.014). Confocal and scanning electron microscopy further confirmed structural disintegration, including bacterial detachment and reduced biofilm thickness. Transcriptome analysis of *S. mutans* biofilm treated with asiatic acid revealed 454 differentially expressed genes (adjusted *p* < 0.05, |log_2_FC| ≥ 1). Notably, genes related to the CiaRH two-component system (*ciaR*, *ciaH*), a central regulatory hub for biofilm maturation and acid tolerance. This disruption initiates a downstream cascade, causing a coordinated downregulation of critical gene clusters essential for virulence and pathogenesis, including stress response (*htrA*, *clpP*, *groEL*, *dnaK*), and the glucan-binding protein gene (*gbpC*) essential for biofilm structural integrity. These findings provide the first mechanistic evidence linking asiatic acid to transcriptional reprogramming in *S. mutans* biofilm, offering a novel ecological strategy for caries prevention by targeting key regulatory pathways.

## 1. Introduction

Dental caries is a biofilm-mediated chronic infectious disease of the oral cavity, affecting approximately 2.4 billion adults and 621 million children [1,2]. Tooth decay arises from biofilms containing cariogenic microorganisms that metabolize fermentable carbohydrates into organic acids. Dental caries can be severe enough to cause pain, pulpal infection, progression to pulpitis and periodontitis, and even tooth loss [3]. Furthermore, dental caries and its associated complications can contribute to or exacerbate systemic disorders, greatly reduce quality of life. These disorders include type I diabetes, inflammatory bowel disease, depression, and several mental disorders [4,5,6,7].

The pathogenesis of caries is inextricably linked to the dysbiosis of oral biofilms, transitioning from a symbiotic microbial community to a pathogenic state dominated by acidogenic and aciduric species [8]. Among various cariogenic bacteria, *Streptococcus mutans*, a Gram-positive facultative anaerobe, is recognized as a key contributor to the biofilms dysbiosis associated with dental caries [9]. Its cariogenicity stems from three core virulence traits: acidogenicity, aciduric properties, and its capacity to assemble an extracellular polymeric matrix in biofilms [9,10]. For acidogenicity, *S. mutans* rapidly ferments dietary carbohydrates (e.g., sucrose, glucose) into lactic acid via the glycolytic pathway, creating a localized pH < 5.5 that demineralizes hydroxyapatite crystals in enamel and dentin [11,12,13]. For aciduricity, the organism thrives in acidic environments (pH 4.0–5.5) through adaptive mechanisms, including the F_1_F_0_-ATPase proton pump and ammonia and polyamine generation pathways, which neutralize intracellular pH and outcompete commensal species [14]. For biofilm formation, *S. mutans* synthesizes extracellular polysaccharides (EPS), extracellular DNA (eDNA), proteins, and lipoteichoic acid, which promotes microbial aggregation and forms spatial structures that restrict diffusion of specific substances and encapsulate bacteria [15]. EPS, particularly dextran, provide binding sites that facilitate bacterial aggregation and attachment. Additionally, they shield microorganisms from desiccation, predation, antimicrobials, antibodies, and bacteriophages. Collectively, these protective functions contribute to the persistence of a localized low-pH microenvironment [16,17]. The biofilm lifecycle begins with the attachment of pioneer colonizers through hydrophobic interactions, calcium bridges, and lectin binding. Secondary colonizers, such as *S. mutans* and *Fusobacteria*, co-aggregate with pioneers, driven by sucrose availability and acidic pH (3.8–4.8). EPS production then forms a three-dimensional matrix that traps acidogenic bacteria (e.g., *Lactobacillus* spp.) and restricts diffusion of saliva, perpetuating a low-pH microenvironment [11,18,19,20]. This acidic niche selectively enriches acid-tolerant pathogens while suppressing health-associated species like *Streptococcus sanguinis* and *Actinomyces naeslundii*, thereby driving ecological catastrophe [21]. Critically, the biofilm’s metabolic heterogeneity allows *S. mutans* to enter a metabolically dormant state under nutrient deprivation, enhancing its survival and recurrence post-treatment.

Despite advances in understanding caries pathogenesis, current therapeutic strategies inadequately target biofilm resilience. Fluoride, the cornerstone of caries prevention, promotes enamel remineralization but fails to inhibit EPS synthesis or biofilm acidogenesis [22]. Chlorhexidine, a broad-spectrum antimicrobial, disrupts microbial diversity and induces resistance in *S. mutans* through efflux pump upregulation [16]. Probiotics like *Lactobacillus rhamnosus* transiently suppress cariogenic species but lack long-term efficacy due to poor colonization [23]. Nanomaterials (e.g., silver nanoparticles) and CRISPR-Cas9-based antimicrobials, though promising, face unresolved safety concerns and regulatory hurdles [24]. These limitations highlight an urgent need for biofilm-disrupting agents that specifically attenuate virulence without perturbing commensal microbiota.

Natural compounds, due to their multi-target mechanisms, low toxicity, and compatibility with oral microbiota, have become ideal candidates to fill this treatment gap [25]. For instance, polyphenols like epigallocatechin gallate (EGCG) has been demonstrated to suppress virulence genes (e.g., *gtfB*, *gbpC*) linked to biofilm formation in *S. mutans* without compromising bacterial viability, as evidenced by its selective inhibition of glucosyltransferase activity and EPS synthesis [26]. This ecological approach aligns with the “antivirulence” paradigm, which restores ecological balance by targeting virulence without disrupting commensals. In the course of studying the anti-biofilm activities of various compounds against *S. mutans*, we observed that asiatic acid (2α,23-dihydroxyursolic acid) exhibits anti-biofilm activity against this bacterium. Asiatic acid, a pentacyclic triterpenoid from Centella asiatica, demonstrates broad-spectrum bioactivity, including anti-inflammatory, antioxidant, and antimicrobial effects [27]. Previous studies have indicated that asiatic acid prevents uropathogenic *Escherichia coli* from adhering to epithelial cells by altering bacterial morphology and the expression of virulence-related genes [28]. Nevertheless, the antibiofilm property of asiatic acid against *S. mutans* and potential mechanisms have rarely been examined.

In this study, we aimed to elucidate the anti-biofilm mechanism of asiatic acid against *S. mutans*, focusing on its role in suppressing cariogenic virulence and biofilm resilience. Utilizing static biofilm models, we quantified the inhibitory effects on acid production, EPS synthesis, and bacterial adhesion asiatic acid. High-resolution imaging and transcriptomic profiling were integrated to characterize biofilm structural disintegration and identify key metabolic pathways. Furthermore, reverse transcription quantitative polymerase chain reaction (RT-qPCR) validation confirmed the downregulation of virulence regulators (e.g., *ciaRH*, *gbpC*) and stress-response genes (e.g., *htrA*, *clpP*). These findings reveal the capacity of asiatic acid to disrupt biofilm maturation through transcriptional reprogramming, positioning it as a novel ecological agent for caries prevention.

## 2. Results

### 2.1. Asiatic Acid Exhibited Selective Anti-Biofilm Activity Against S. mutans

**Figure 1:**
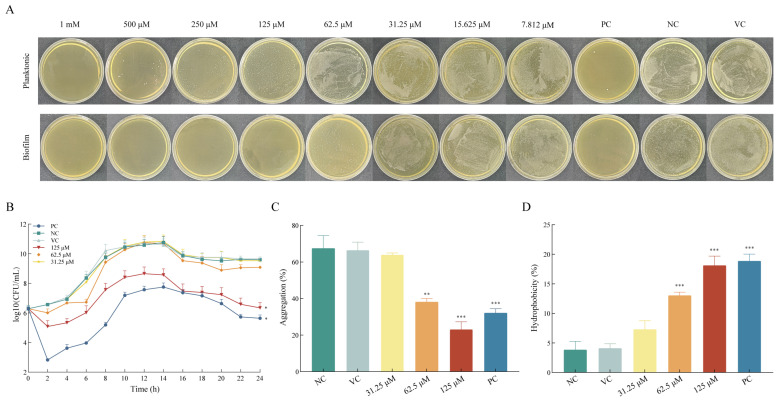
The minimum biofilm inhibitory concentration and antibacterial activity of asiatic acid for *S. mutans*. (**A**) Minimum biofilm inhibitory concentration (MBIC) and inhibitory concentration (MIC) of asiatic acid for *S. mutans*, (**B**) growth curves, (**C**) auto-aggregation and (**D**) surface hydrophobicity of *S. mutans*. PC: positive control (0.12% CHX); NC: negative control (saline); VC: vehicle control (1.25% DMSO). * *p* < 0.05 compared to NC. ** *p* < 0.01 compared to NC. *** *p* < 0.001 compared to NC.

Asiatic acid exhibited potent biofilm inhibition against *S. mutans* with the MBIC of 62.5 μM, five-fold lower than its MIC for planktonic growth (500 μM) (Figure 1A). Consequently, 31.25, 62.5, and 125 μM asiatic acid were selected for further experiments. Growth curve analysis revealed that 0.12% CHX and 125 μM asiatic acid effectively reduced bacterial counts within 2 h, with CHX exhibiting a more pronounced effect (Figure 1B). In contrast, at 62.5 μM asiatic acid transiently delayed bacterial proliferation but permitted regrowth by 8 h, confirming its non-bactericidal mode of action at MBIC levels. Together, the MBIC (62.5 μM) delayed bacterial proliferation without bactericidal effects, indicating asiatic acid’s biofilm-specific inhibitory activity.

### 2.2. Asiatic Acid Decreased Adhesion of S. mutans

As illustrated in Figure 1C, the negative control group exhibited high potential aggregation, while treatment with asiatic acid significantly impaired the auto-aggregation of *S. mutans*. Specifically, at concentrations of 62.5 and 125 μM, aggregation was observed to be 38.07 ± 2.67% and 22.94 ± 6.15% (both *p* < 0.005), respectively. Moreover, the surface hydrophobicity ability of *S. mutans* with 62.5 and 125 μM of asiatic acid was increased to 13.00 ± 1.19% and 18.06 ± 3.25% (both *p* < 0.001), respectively (Figure 1D). Together, these dual effects correlated with diminished sucrose-dependent biofilm cohesion.

### 2.3. Asiatic Acid Suppressed Biofilm Formation of S. mutans

**Figure 2:**
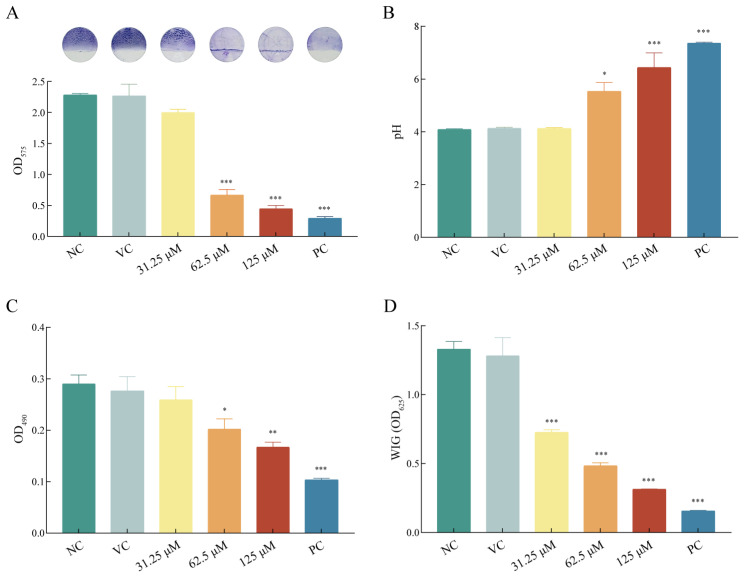
Anti-biofilm activity of asiatic acid for *S. mutans*. (**A**) Crystal violet staining, (**B**) pH value, (**C**) Metabolic activity and (**D**) Extracellular insoluble glucans (WIG) content of *S. mutans* biofilms. PC: positive control (0.12% CHX); NC: negative control (saline); VC: vehicle control (1.25% DMSO). * *p* < 0.05 compared to NC. ** *p* < 0.01 compared to NC. *** *p* < 0.001 compared to NC.

To investigate the sucrose-dependent adhesion biofilm of *S. mutans*, we utilized the AAA model to assess changes in biomass, acid production, metabolic activity, and EPS content following 24 h of asiatic acid treatment. Asiatic acid treatment caused a dose-dependent reduction in biofilm biomass. As shown in Figure 2A, quantitative analysis of the crystal violet staining revealed that treatment with 62.5 and 125 μM asiatic acid substantially reduced the biofilm biomass of *S. mutans* (OD_575nm_ < 0.9, both *p* < 0.001). Morphologically, biofilms treated with ≥62.5 μM appeared translucent with sparse bacterial clusters at the air-liquid interface. Treatment with 62.5 μM asiatic acid elevated the biofilm microenvironment pH from 4.09 ± 0.06 to 5.54 ± 0.67 (*p* = 0.017), while 125 μM achieved near-neutral pH 6.44 ± 1.11 (*p* < 0.001) (Figure 2B). This pH shift exceeded the critical threshold for enamel demineralization (pH 5.5), suggesting that asiatic acid attenuated acidogenic capacity of *S. mutans*. Asiatic acid also effectively reduced metabolic activity of *S. mutans* biofilm. The results presented in Figure 2C indicate that the OD values at 490 nm for the 62.5 and 125 μM of asiatic acid, recorded at 0.19 ± 0.02 (*p* = 0.014) and 0.17 ± 0.02 (*p* = 0.002), respectively. The non-linear dose–response relationship indicated saturation of metabolic inhibition above MBIC. For extracellular water insoluble glucans (WIG), the WIG of *S. mutans* biofilms treated with 62.5 and 125 μM of asiatic acid were both lower than that of the positive control group (Figure 2D). The OD values for WIG at 625 nm in the treatment groups of 31.25 μM, 62.5 μM, and 125 μM were recorded as 0.73 ± 0.03, 0.48 ± 0.04, and 0.31 ± 0.00 (all *p* < 0.001), respectively.

### 2.4. Asiatic Acid Altered Structural Morphology of S. mutans Biofilms

**Figure 3:**
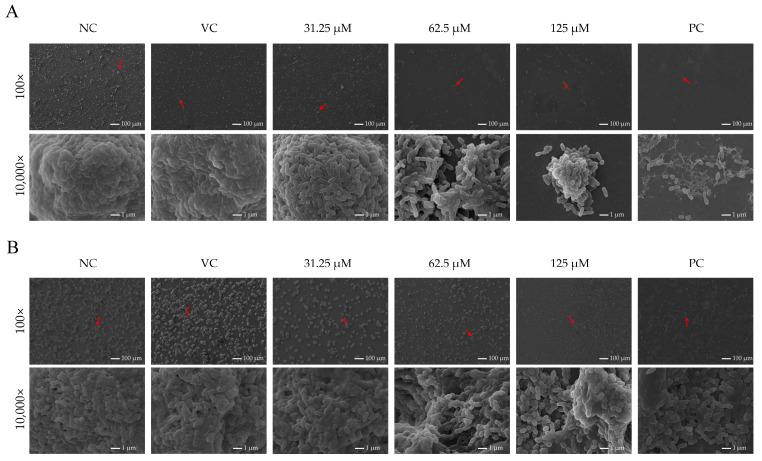
SEM images of *S. mutans* biofilm exposed to asiatic acid. (**A**) Biofilm formation, (**B**) mature biofilm. Red arrows point to the field of view at 10,000× magnification. PC: positive control (0.12% CHX); NC: negative control (saline); VC: vehicle control (1.25% DMSO).

SEM and CLSM were employed to observe the architectural disruption of *S. mutans* biofilms induced by asiatic acid. In contrast to the dense and robust biofilm formed by the negative control groups (Figure 3A), treatment with asiatic acid triggered significant architectural disruption. At 31.25 μM, asiatic acid triggered partial bacterial detachment and extracellular matrix (ECM) fragmentation (Figure 3A). Whereas, in the 62.5 and 125 μM asiatic acid-treated groups, the colonies were sparsely distributed and the ECM was significantly reduced. For mature biofilm, untreated biofilms formed stratified architectures with thick ECM layers (Figure 3B). Asiatic acid (62.5 and 125 μM) induced “highway overpass”-like cavities connecting isolated microcolonies, accompanied by complete ECM dissolution.

**Figure 4:**
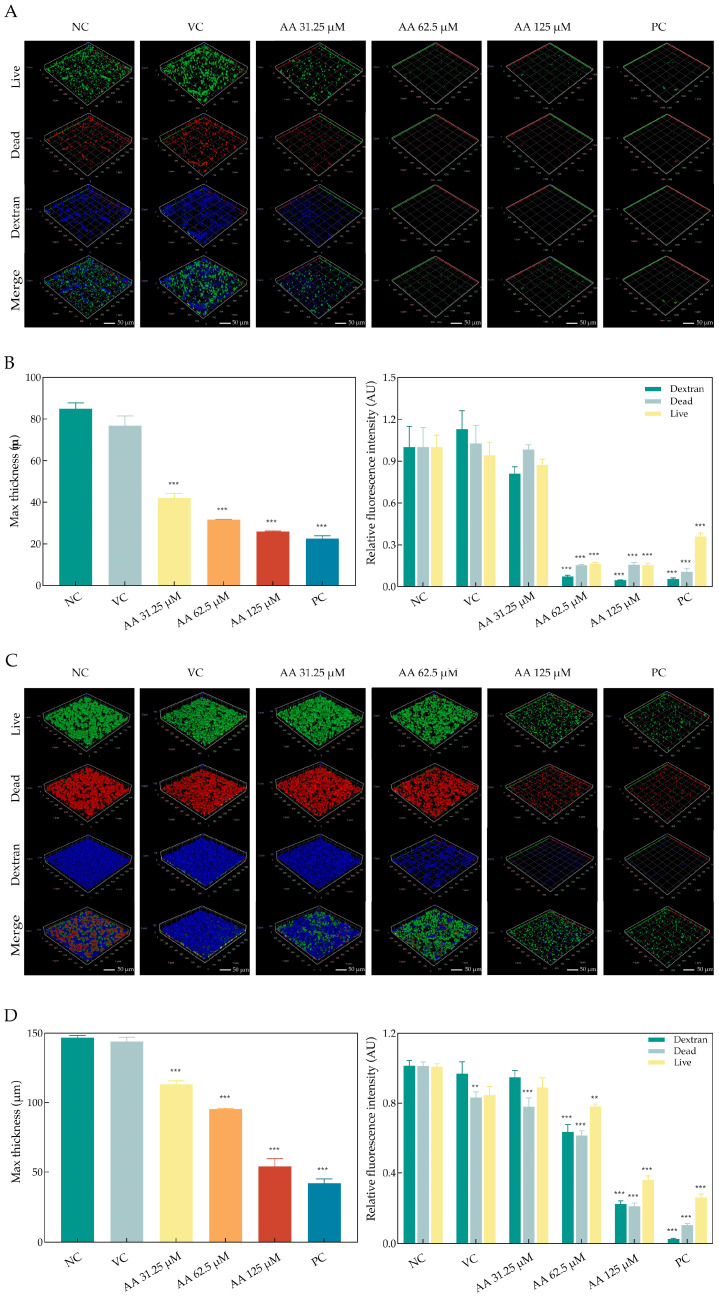
3D images of *S. mutans* biofilm exposed to asiatic acid as observed by CLSM. (**A**,**C**) Merged image of live/dead staining and EPS staining of the biofilms exposed to asiatic acid after 24 h and 48 h, respectively. (**B**,**D**) Thickness identified by Z- axis length, and the relative fluorescence intensity (RFI) of SYTO 9/PI and Alexa Fluor 647 of *S. mutans* biofilms exposed to asiatic acid after 24 h and 48 h, respectively. PC: positive control (0.12% CHX); NC: negative control (saline); VC: vehicle control (1.25% DMSO). ** *p* < 0.01 compared to NC. *** *p* < 0.001 compared to NC.

Meanwhile, the CLSM microscope images unveiled that asiatic acid’s biphasic disruption of *S. mutans* biofilms—initially suppressing nascent matrix assembly during formative stages (24 h), then eroding pre-established architectures in mature communities (48 h). The relative average fluorescence intensity of images was utilized to assess the number of live and dead bacteria and the EPS content. After 24 h of treatment, untreated biofilms exhibited dense, confluent layers of viable bacteria (intense green SYTO 9 fluorescence) embedded within a continuous EPS matrix (blue Alexa Fluor 647 signal), with minimal cell death (low red PI fluorescence, Figure 4A). In contrast, asiatic acid treatment resulted in a significant reduction in live bacterial clusters and a concomitant increase in dead cells. At 62.5 μM, biofilms displayed scattered microcolonies of live cells, reduced EPS continuity, and increased porosity. At 125 μM, live bacteria were sparse and isolated within a highly fragmented EPS matrix, accompanied by substantial regions of PI staining indicating loss of viability. Quantitative analysis confirmed a reduction in maximum biofilm thickness from 84.94 ± 4.82 μm (negative control) to 25.99 ± 0.42 μm at 125 μM asiatic acid (*p* < 0.001). The relative fluorescence intensity (RFI) of SYTO 9 decreased by 64%, while PI intensity increased by 79%. EPS signal (Alexa Fluor 647) declined by 78% relative to the negative control (Figure 4B, Table S3). In mature 48 h biofilms, mature biofilms in the negative control group showed thickened, multilayered architectures with consolidated viable clusters and robust EPS encapsulation (Figure 4C). Asiatic acid induced severe structural collapse: at 62.5 μM, biofilm thickness decreased to 95.33 ± 1.15 μm, with visible cavities and reduced SYTO 9 intensity. At 125 μM, maximum thickness was further reduced to 54.11 ± 9.46 μm (*p* < 0.001). The RFI of SYTO 9 decreased to 15% of the negative control levels with a concomitant 84% reduction in PI intensity. The EPS matrix was reduced to 5% of control RFI (Figure 4D, Table S4). Three-dimensional reconstructions illustrated widespread disintegration of biofilm integrity, with large hollow regions and loss of structural continuity.

### 2.5. Transcriptomic Profiling Reveals Key Pathways Modulated by Asiatic Acid

**Figure 5:**
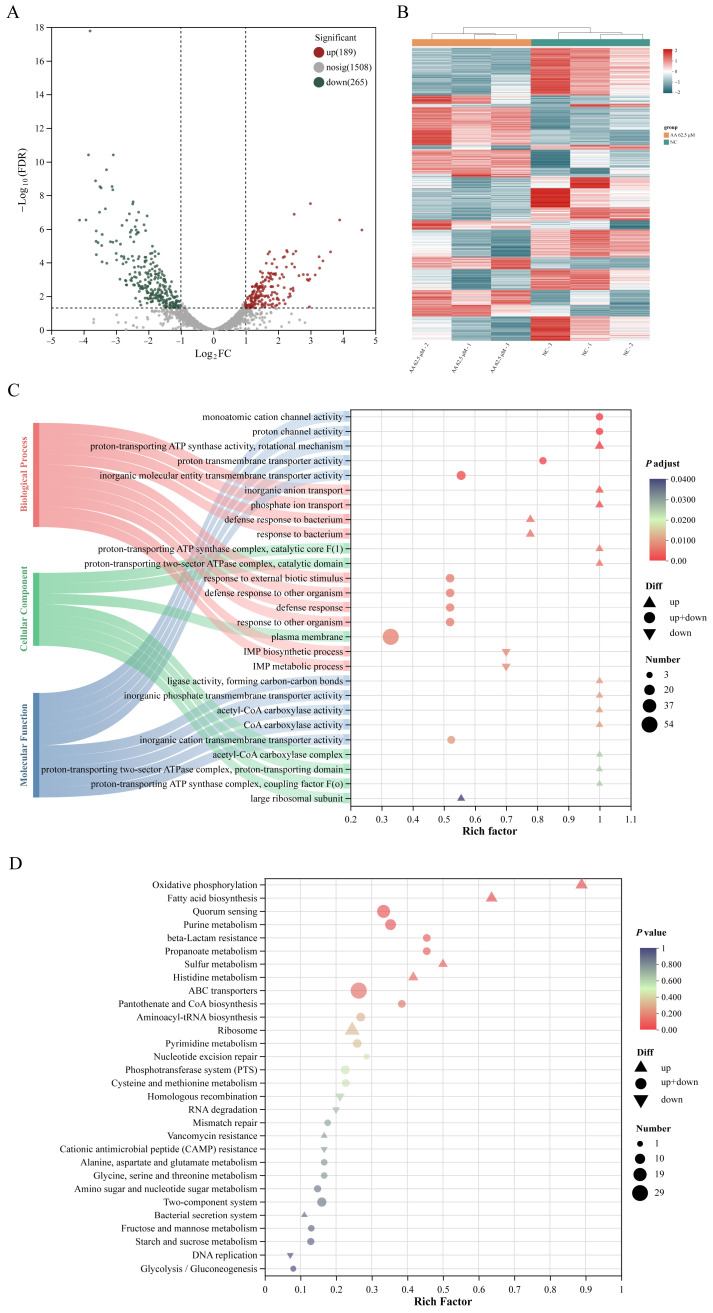
Transcriptome analysis of asiatic acid on the *S. mutans* biofilm. (**A**) Volcano plot showing differentially expressed genes (DEGs) from RNA-seq data. Genes shown in red are significantly upregulated with a fold change greater than 2 and *p*-value < 0.05, followed by genes in blue are significantly downregulated. (**B**) Heat map representation of DEGs between samples. (**C**) GO enrichment cluster analyses and (**D**) KEGG enrichment cluster analyses of the differentially expressed genes.

Asiatic acid (62.5 μM) triggered transcriptional reprogramming in *S. mutans* biofilms, yielding 454 DEGs (adjusted *p* < 0.05 and |log_2_FC| ≥ 1) with 265 downregulated and 189 upregulated (Figure 5A). K-means clustered heatmap indicated stark divergence between treated and control transcriptomes (Figure 5B), aligning with phenotypic suppression of biofilm formation.

To categorize the biological roles of these DEGs, we performed GO and KEGG enrichment analyses. GO analysis indicated that downregulated genes were significantly enriched in terms related to nucleotide metabolism, while upregulated genes were associated with ion transport and defense responses (Figure 5C). The KEGG analysis further highlighted that key downregulated genes were concentrated in pathways crucial for virulence, including quorum sensing (QS), two-component system (TCS),, and purine metabolism (Figure 5D).

**Figure 6:**
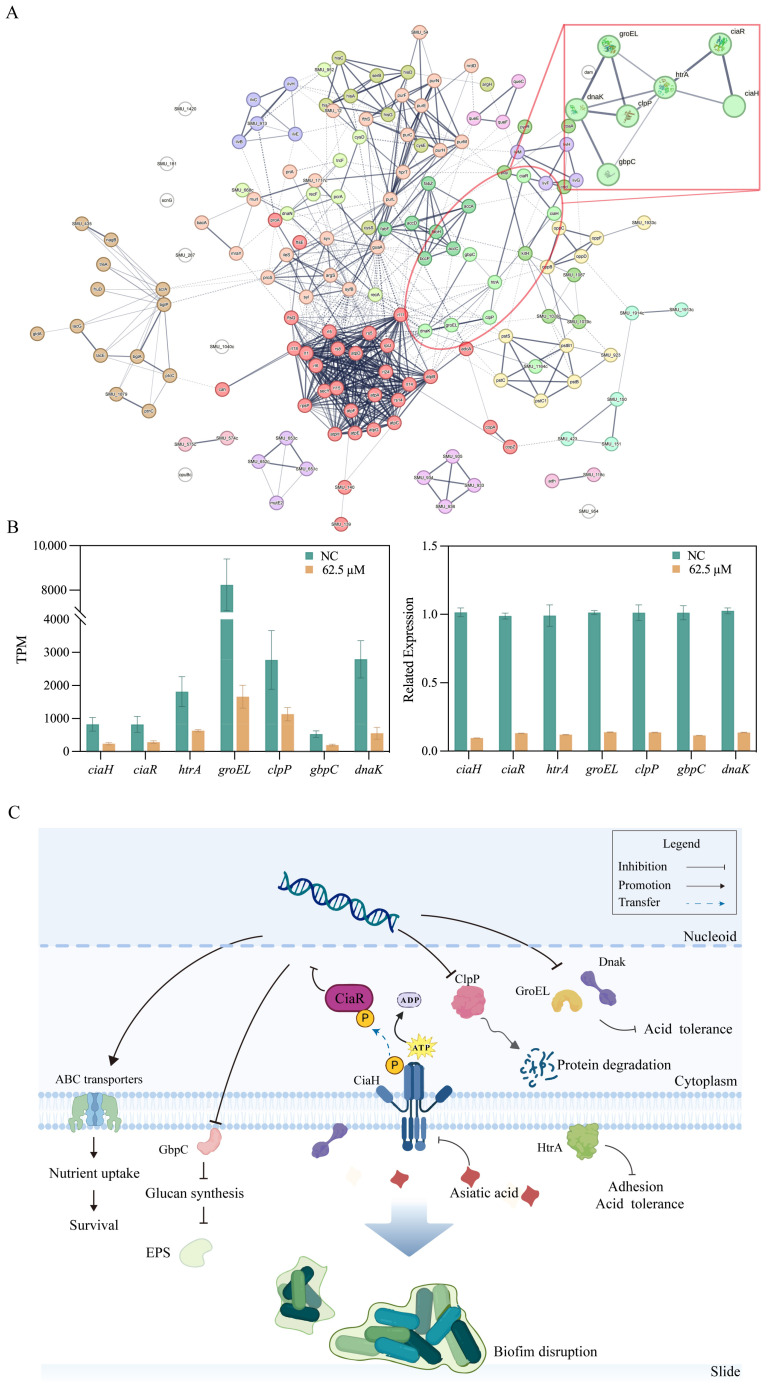
Potential regulators of *S. mutans* biofilm formation inhibited by asiatic acid. (**A**) The protein–protein interaction (PPI) network of differentially expressed genes (DEGs) clustered in KEGG enrichment. (**B**) RT-qPCR (right) examined the relative mRNA levels (left) of 7 related to biofilm formation. (**C**) Critical regulatory role of the quorum sensing system in *S. mutans* biofilms.

To pinpoint the core regulatory network within these affected pathways, we then performed a protein–protein interaction (PPI) network analysis. Protein–protein interaction (PPI) network analysis of the KEGG-enriched DEGs comprising 156 nodes and 1351 edges. Among the DEGs, we identified 17 gene clusters (*p* < 0.05) that were significantly enriched in processes related to metabolic pathways, oxidative phosphorylation, QS, and the ABC transporter pathway (Figure 6A and Table S1). Notably, this cluster included genes encoding the CiaRH two-component system (*ciaR*, *ciaH*), essential stress response proteins (*htrA*, *groEL*, *clpP*, *dnaK*), and the critical glucan-binding protein GbpC. This finding identifies a specific virulence-associated genetic network as a primary target of asiatic acid.

### 2.6. RT-qPCR Confirms Downregulation of Biofilm-Associated Genes

To validate the transcriptomic findings and confirm the impact of asiatic acid on pivotal genes associated with *S. mutans* biofilm formation and virulence, RT-qPCR was performed. Seven DEGs from Cluster 8 of the PPI network were selected for validation. These genes—*ciaH*, *ciaR*, *htrA*, *groEL*, *clpP*, *gbpC*, and *dnak* are central to quorum sensing, stress response, and EPS production. The RT-qPCR results demonstrated a consistent pattern of downregulation for all seven selected genes in the presence of asiatic acid, corroborating the RNA-sequencing data and reinforcing the reliability of the transcriptomic analysis (Figure 6B and Table S2). Specifically, the relative mRNA expression levels of genes encoding the CiaRH two-component system components, *ciaH* and *ciaR*, were significantly reduced. Similarly, genes involved in stress response and protein quality control, such as *htrA* (serine protease A), *groEL* (chaperonin), *clpP* (ATP-dependent Clp protease proteolytic subunit), and *dnak* (heat shock protein 70), also exhibited marked downregulation upon asiatic acid treatment. Furthermore, the expression of *gbpC* (glucan-binding protein C), which plays a role in dextran-dependent aggregation and biofilm structure, was also significantly suppressed. These targeted RT-qPCR analyses confirm that asiatic acid, at its MBIC, effectively represses the transcription of key genes essential for *S. mutans* biofilm development, stress tolerance, and virulence, providing further mechanistic insight into its anti-biofilm activity.

## 3. Discussion

The rising challenge of dental caries demands novel approaches targeting the virulence of key pathogens like *S. mutans*. This study demonstrates that asiatic acid, a natural pentacyclic triterpenoid, exhibits potent anti-biofilm activity against *S. mutans* at concentrations that do not inhibit planktonic growth, highlighting its potential as a targeted anti-virulence agent rather than a broad-spectrum antimicrobial. Our findings reveal that asiatic acid’s efficacy stems from its multifaceted disruption of key cariogenic processes, including adhesion, EPS production, acidogenesis, and metabolic activity, ultimately leading to compromised biofilm integrity. A critical aspect of *S. mutans* cariogenicity is its ability to form robust biofilms. Asiatic acid significantly impaired initial bacterial adhesion, as evidenced by reduced auto-aggregation and altered surface hydrophobicity. This early disruption likely hinders the foundational stages of biofilm development. Furthermore, the substantial reduction in EPS, particularly water-insoluble glucans which form the structural scaffold of the biofilm matrix, corroborates the observed loosening and thinning of the biofilm structure in SEM and CLSM analyses. The three-dimensional imaging revealed a progressive dismantling of architectural integrity by asiatic acid—from porous nascent matrices to cavity-riddled mature structures. During initial formation, asiatic acid appears to preferentially target nascent EPS synthesis to prevent biofilm consolidation, while in established communities, it effectively dismantles the pre-established ECM to destabilize mature biofilms. This mode of action suggests a targeted disruption of virulence without compromising structural homeostasis of the bacterial cells themselves. By diminishing EPS, asiatic acid not only weakens the biofilm’s physical structure but may also increase its susceptibility to other antimicrobial agents or host defenses [29]. Beyond structural integrity, the acidogenic capacity is a critical virulence factor contributing to dental caries caused by S. mutans. Our results show that asiatic acid effectively neutralized the biofilm microenvironment, raising the pH to levels above the critical threshold for enamel demineralization. As the concentration of asiatic acid increased, the levels of EPS in the biofilm decreased correspondingly, with a near-complete elimination of WIG at 125 µM that correlated with the observed structural collapse. The combined suppression of biofilm biomass, acidogenesis, metabolism, and structural polysaccharides demonstrates asiatic acid’s multi-faceted anti-biofilm action, highlighting its potential as an ecological caries prophylactic agent.

The transcriptomic analysis provided profound insights into the molecular mechanisms underpinning asiatic acid’s anti-biofilm effects. A significant number of DEGs were identified, with a notable downregulation of genes crucial for virulence and biofilm homeostasis. Our findings showed that the downregulation of genes involved in the CiaRH two-component system (*ciaR*, *ciaH*) is particularly significant. The CiaRH system is a pivotal regulator in *S. mutans*, influencing biofilm formation, acid tolerance, genetic competence, and bacteriocin production [30]. The genes *ciaH* and *ciaR* encode a histidine kinase sensor protein and its cognate response regulator [31]. As reported in previous studies, Environmental stimulation results in the autophosphorylation of CiaH, which then activates CiaR. Subsequently, activated CiaR binds to the direct repeats upstream, enhancing transcription of the *ciaRH* operon. The absence of *ciaH* alters sucrose-dependent biofilm formation, abolished mutacin production, diminished competence development and increased sensitivity to acidic pH [32,33]. Lévesque et al. validated that deletion of CiaH resulted in mutant biofilms with reduced biomass and shorter chains [34]. Disruption of CiaRH signaling, as suggested by our data, likely contributes to the observed phenotypic changes, including reduced biofilm biomass and altered structure. The observed downregulation of *ciaRH* provides strong evidence for this pathway being a key target of asiatic acid. This targeted effect on a central regulatory hub represents a sophisticated mechanism for attenuating *S. mutans* virulence.

Moreover, asiatic acid treatment led to the downregulation of several stress response genes, including *htrA*, *clpP*, *groEL*, and *dnaK*. These genes encode chaperones and proteases that are essential for maintaining protein homeostasis and enabling survival under stressful conditions, such as the acidic environment within mature biofilms [35]. In addition, CiaH signal transduction may be linked with the surface-anchored serine-protease HtrA, which functions as both a molecular chaperone and a proteases [36,37]. By suppressing these protective mechanisms, asiatic acid may render *S. mutans* more vulnerable within the biofilm niche and impair its ability to adapt to environmental challenges, aligning with the observed reduction in metabolic activity and acid tolerance. This topology the PPI network analysis supports this interpretation. Asiatic acid’s suppression of CiaRH disrupted upstream signaling cascades, while coordinated downregulation of *dnaK*, *groEL* and *clpP* crippled bacterial capacity to counteract metabolic stress. This dual-pronged attack on biofilm stability. GbpC, a key cell-surface-associated protein, is implicated in the adherence and dextran-induced aggregation of *S. mutans* and is expressed only under stress conditions [38]. Notably, GbpC interacts with GtfD to produce dextran, facilitating sucrose-dependent adhesion [39]. The downregulation of *gbpC* directly explains the reduced EPS content and further supports the anti-biofilm phenotype. The highly specific transcriptional reprogramming centered on the CiaRH system, observed in the absence of broad cytotoxicity, strongly suggests a targeted mechanism of action rather than general membrane disruption. While our study does not provide direct evidence of a physical interaction, the transcriptomic data are consistent with a model where asiatic acid modulates the CiaH sensor kinase. We hypothesize that the amphipathic structure of asiatic acid allows it to partition into the cell membrane, where it could allosterically interfere with CiaH function. Such interference could impede kinase autophosphorylation, alter its conformation, or disrupt protein–protein interaction, thereby blocking downstream signal transduction. This proposed mechanism provides a compelling, testable framework for future biochemical and structural investigations.

Our results revealed that asiatic acid as a non-canonical kinase antagonist, inhibits CiaRH activation, disrupts TCS-mediated stress regulation, and ultimately collapses exopolysaccharide biosynthesis (Figure 6C). Interestingly, while virulence-related pathways were largely suppressed, genes associated with ABC transporters and certain amino acid metabolism pathways were upregulated. This could represent a compensatory stress response, where the bacteria attempt to enhance nutrient uptake or adapt their metabolism to survive the challenging conditions imposed by asiatic acid [40]. This dichotomy mirrors an evolutionary trade-off—sacrificing pathogenicity to prioritize survival under metabolic duress, highlighting a complex bacterial response and warrants further investigation. It suggests that while asiatic acid effectively cripples pathogenic traits, *S. mutans* may still attempt to persist, emphasizing the need for strategies that can overcome such adaptive responses.

Our work builds upon and significantly extends previous findings. While earlier studies focused on the general antimicrobial properties of natural compounds [27,41], and one report noted the inhibitory phenotype of asiatic acid on *S. mutans* biofilms [42], our study provides the first in-depth mechanistic investigation at the transcriptomic level. The detailed transcriptomic profile, coupled with phenotypic validation, offers a more comprehensive picture than previously available, pinpointing specific molecular targets such as the CiaRH system and key stress response effectors. The findings suggest that asiatic acid acts not as a bactericidal agent at its MBIC but as an ecological modulator, attenuating the pathogenic potential of *S. mutans*.

This anti-virulence approach is gaining traction as a strategy to control infections while minimizing the risk of resistance development and disruption to the commensal microbiota.

Although our study offers important insights into the anti-biofilm efficacy of asiatic acid, its limitations indicate valuable directions for further investigation. Our findings were obtained using a well-defined monospecies model, which, while useful for mechanistic analysis, does not fully replicate the complexity of natural oral biofilms. Extending this work to multispecies or saliva-derived biofilms would help further ascertain asiatic acid’s broader applicability and ecological impact. In human cell lines, asiatic acid exhibited minimal cytotoxicity against human oral cells, with a cell viability rate of 88.6% [43]. In addition, the current evidence is based entirely on in vitro assays; thus, validation in vivo models—such as rodent caries experiments—would strengthen the translational relevance of our results. Subsequent comprehensive clinical trials are necessary to establish a safe and effective dosage for oral health applications in humans, ensuring that local concentrations do not adversely affect oral mucosal cells. Finally, the potential for bacterial adaptation under prolonged exposure warrants further investigation through serial passaging and resistance induction assays. Beyond these limitations, a deeper understanding of asiatic acid’s molecular mechanism—specifically its binding to the CiaH protein—and its synergy with other anticaries agents are critical steps for its future translational development.

## 4. Materials and Methods

### 4.1. Bacterial Strain and Culture Conditions

*S. mutans* UA159 was stored at −80 °C and routinely cultured in brain heart infusion (BHI) broth (Thermo Fisher Scientific, Waltham, MA, USA) under anaerobic conditions (10% H_2_, 5% CO_2_, 85% N_2_) at 37 °C. For biofilm formation and mature biofilm, *S. mutans* was cultured in BHI broth supplemented with 1% (*w*/*v*) sucrose (BHIS, Macklin, Shanghai, China). Asiatic acid with a purity of 98% (Solarbio, Beijing, China) was dissolved in dimethyl sulfoxide (DMSO, Aladdin, Shanghai, China) to prepare a stock solution. Controls included 0.12% chlorhexidine digluconate (CHX, Macklin, Shanghai, China) as positive control, saline (Sigma-Aldrich, Shanghai, China) as negative control, and 1.25% DMSO as vehicle control.

### 4.2. Minimal Inhibitory Concentration

The antibacterial and anti-biofilm activities of asiatic acid were evaluated via colony-forming unit (CFU) assay. For planktonic growth inhibition, overnight cultures (approximately 10^8^ CFU/mL) were incubated with asiatic acid ranging from 1000 to 7.812 μM in 24-well plates for 24 h under stationary conditions. The lowest concentration preventing planktonic CFU increase was defined as the minimum inhibitory concentration (MIC). Biofilm inhibition was assessed using the Amsterdam active attachment (AAA) model [22]: glass slides pre-coated with artificial saliva were vertically immersed in bacterial suspensions containing test agents, followed by anaerobic incubation for 24 h. Biofilms were harvested, homogenized in saline, serially diluted, and plated to determine the minimum biofilm inhibitory concentration (MBIC). The MBIC represents the minimum concentration required to suppress biofilm formation without compromising planktonic viability, while stabilizing viable cell counts within the biofilm [44].

### 4.3. Planktonic Growth Dynamics

The antibacterial activity of asiatic acid against planktonic *S. mutans* was evaluated using a growth curve assay. Overnight cultures were centrifuged, resuspended in fresh BHI broth (1–5 × 10^6^ CFU/mL), and treated with asiatic acid (31.25, 62.5, 125 μM). Bacterial growth was monitored every 2 h during anaerobic incubation at 37 °C for 24 h using CFU enumeration. Sample collection for CFU counting was performed aseptically inside an anaerobic chamber to prevent disturbance of the anaerobic conditions.

### 4.4. Bacterial Auto-Aggregation

The auto-aggregation of bacterial cells was assessed as previously described with minor modifications [45]. Briefly, an overnight culture of *S. mutans* was adjusted to an optical density (OD) of 0.5 at 600 nm using phosphate-buffered saline (PBS, Sigma-Aldrich, Shanghai, China) supplemented with 1% (*w*/*v*) sucrose. The initial OD_600nm_ was recorded, and the samples were incubated with asiatic acid (31.25, 62.5, and 125 μM) at 37 °C for 2 h in stationary condition. After incubation, the OD_600nm_ of the supernatant was measured. The rate of aggregation was calculated by the following equation: Aggregation (%) = (OD_Initial_ − OD_2h_)/(OD_Initial_ − OD_Blank_) × 100%.

### 4.5. Bacterial Surface Hydrophobicity

Bacterial surface hydrophobicity was assessed based on microbial adherence to hydrocarbons, as described by He et al. [33] with minor modifications. Briefly, bacterial suspensions were incubated with asiatic acid for 30 min, followed by addition of xylene (20% *v*/*v*, Aladdin, Shanghai, China), vigorous mixing, and phase separation. The optical density of the aqueous phase (OD_2_) and initial mixture (OD_1_) at 550 nm was measured. The percentage of hydrophobicity was calculated using the following equation: H = (OD_1_ − OD_2_)/OD_2_ × 100%. The final hydrophobicity rate was determined as the difference between values at 0 min (H_1_) and 30 min (H_2_).

### 4.6. Biofilm Characterization

#### 4.6.1. Biomass

The crystal violet (CV) staining assay was employed to quantitatively assess biofilm biomass in the AAA model [46]. In brief, the attached biofilm was fixed with methanol for 15 min, and then stained with 0.1% (*v*/*v*) CV (Solarbio, Beijing, China) for 10 min. The excess CV solution was carefully removed, and the slides were rinsed thoroughly with sterile water until the wells appeared colorless. After drying, and 33% glacial acetic acid solution (Macklin, Shanghai, China) was added to solubilize the bound CV for 10 min. Absorbance was measured at 575 nm to quantify biofilm formation.

#### 4.6.2. Acid Production

After the incubation for *S. mutans* biofilm, the pH of the spent medium was measured using a precise pH meter (PHS-2C, Mettler Toledo, Shanghai, China).

#### 4.6.3. Metabolic Activity

The MTT [3-(4,5-dimethylthiazol-2-yl)-2,5-diphenyltetrazolium bromide] assay was utilized to evaluate the metabolic activity of viable biofilm cells [47]. Biofilms were suspended in 1 mL PBS, and dispersed by vortexing for 30 s, followed by low-temperature sonication for 10 min. The biofilm cells were then concentrated to 100 µL and transferred to a 96-well plate. Each well incubated with 5 mg/mL MTT solution (Solarbio, Beijing, China) at 37 °C for 4 h in the dark. Formazan crystals (Solarbio, Beijing, China) were dissolved in 110 μL DMSO, and absorbance was quantified at 490 nm.

#### 4.6.4. Exopolysaccharides (EPS)

The EPS of *S. mutans* biofilms were measured using the anthrone-sulfuric acid method [48]. The biofilm suspension was centrifuged at 6000× *g* at 4 °C for 10 min to harvest the supernatant, which contained water-insoluble glucans (WIG). Water-soluble glucans (WSG) in the precipitate were extracted using 0.8 mol/L NaOH (Fengchuan, Tianjin, China) with agitation for 2 h at 37 °C, followed by centrifugation. Anthrone-sulfuric acid reagent (3:1 *v*/*v*) was added, followed by heating (96 °C, 6 min), cooling, and absorbance quantification at 625 nm.

### 4.7. Biofilm Structural Imaging

#### 4.7.1. Scanning Electron Microscopy (SEM)

The microstructure of *S. mutans* biofilm and the distribution of bacteria were observed as previously described [49]. The biofilms were fixed with 2.5% (*v*/*v*) glutaraldehyde overnight at 4 °C in the dark, and dehydrated through serial concentrations of ethanol (30%, 40%, 50%, 60%, 70%, 80%, 90%, and 100%, *v*/*v*) for 15 min each. The slides with the attached biofilms were critical-point dried, sputter-coated with gold, and imaged using the SEM (JSM-IT300LV, JEOL, Tokyo, Japan) at 100× and 10,000× magnifications.

#### 4.7.2. Confocal Laser Scanning Microscopy (CLSM)

To analyze the structure and viability of *S. mutans* biofilm, CLSM (LSM 980, Carl Zeiss, Shanghai, China) was utilized for observing live/dead cells and EPS staining, as previously described methods with slight modifications [50]. For live/dead staining: SYTO 9 (495/515 nm, green, Thermo Fisher Scientific, Waltham, MA, USA) and propidium iodide (PI, 528/617 nm, red, Thermo Fisher Scientific, Waltham, MA, USA) were applied for 15 min in the dark. Dextran was labeled with Alexa Fluor 647 (650/668 nm, blue, Thermo Fisher Scientific, Waltham, MA, USA). Z-axis was used to record the biofilm thickness, and five randomly selected positions were scanned at 10× magnification. In each experiment, the excitation laser intensity, background level, contrast, and electronic zoom were maintained at the same level.

### 4.8. Transcriptomic Profiling and Molecular Validation

#### 4.8.1. RNA Sequencing and Bioinformatics Analysis

Transcriptome data were obtained from the negative control and the 62.5 μM asiatic acid group, with technical support from Shanghai Majorbio Bio-pharm Technology Co., Ltd. Samples were transported on dry ice to the facility for RNA extraction and raw data acquisition. The raw RNA-seq data for this study are accessible through the NCBI Sequence Read Archive (SRA) under accession number PRJNA1136206. The raw data was processed with custom Perl scripts to obtain clean data, which were then aligned to the *Streptococcus mutans* reference genome (GenBank assembly number GCA_000007465.2). The DESeq2 package was utilized to identify differentially expressed genes (DEGs) based on the following criteria: adjusted *p*-value < 0.05 and |log_2_FC| ≥ 1. Subsequently, DEGs were subjected to Gene Ontology (GO) and Kyoto Encyclopedia of Genes and Genomes (KEGG) pathway analyses via Goatools and KOBAS 2.0 with Fisher’s exact test [51].

#### 4.8.2. Reverse Transcription Quantitative PCR (RT-qPCR)

Seven DEGs (*ciaRH*, *htrA*, *clpP*, *groEL*, *gbpC*, *dnaK*) were validated by SYBR Green RT-qPCR. Primers were designed via Primer-BLAST (NCBI) and synthesized by GENEWIZ Biotechnology Co., Ltd. (Suzhou, China) as detailed in Table S3. The 2^−ΔΔCt^ method was applied to evaluate gene expression level, normalizing to the housekeeping gene (the 16S rRNA gene of *S. mutans*).

### 4.9. Statistical Analysis

All experiments were conducted independently in triplicate. The results are presented as means ± standard deviations and were analyzed using SPSS version 26.0. One-way ANOVA followed by Benjamini–Hochberg (BH) comparison tests was performed at a significance level of *p* < 0.05.

## 5. Conclusions

In conclusion, this study comprehensively demonstrates that asiatic acid effectively inhibits *S. mutans* biofilm formation and attenuates key cariogenic virulence factors at physiologically relevant concentrations. Phenotypic and transcriptomic analyses showed that asiatic acid downregulates key genes for biofilm structure (*gbpC*), acid tolerance, stress response (e.g., *htrA*, *clpP*, *dnak*, *groEL*), and the pivotal CiaRH two-component signaling system. By targeting specific virulence mechanisms rather than bacterial viability, asiatic acid exemplifies a promising anti-virulence strategy. These findings establish asiatic acid as a strong candidate for development as a novel, plant-derived agent for an ecological approach to preventing dental caries. Further in vivo investigations are warranted to translate these promising findings into effective clinical applications.

## Data Availability

In this study, the datasets are available on request to the corresponding author.

## Supplementary Materials

The following supporting information can be downloaded at: https://www.mdpi.com/article/10.3390/ijms26199510/s1.
