# Supplementary material for: Asiatic Acid Disrupts the Biofilm Virulence of Streptococcus mutans by Transcriptional Reprogramming of Quorum Sensing System
Source: Int J Mol Sci. 2025 Sep 29;26(19):9510. doi: 10.3390/ijms26199510 (PMC12525332; doi:10.3390/ijms26199510)
Supplement: Supplementary file 1 [file ijms-26-09510-s001.zip › ijms-3830303-supplementary.pdf]

Supporting information

Asiatic Acid Disrupts the Biofilm Virulence of *Streptococcus mutans* by Transcriptional Reprogramming of Quorum Sensing System

Qingying Shi [1,a], Fengzhu Li [1,3,a], Yingying Peng [1], Qiannan Sun [1], Hong Zhao [2], Congcong Wang [1], Shulin Liu [1], Fuping Lu [1], Huabing Zhao [1,*]

[1] Key Laboratory of Industrial Fermentation Microbiology, Ministry of Education, Tianjin Key Laboratory of Industrial Microbiology, College of Biotechnology, Tianjin University of Science and Technology, 9 TEDA 13th Street, Tianjin 300457, China

[2] Tianjin Customs Animal, Plant, and Food Testing Center, No. 51, Second Avenue, Tianjin Economic-Technological Development Area (TEDA), Binhai New Area, Tian-jin 300457, China

[3] State Key Laboratory of Food Science and Resources, Nanchang University, No. 235 Nanjing East Road, Nanchang, 330031, China

[a] Authors contributed equally.

* Correspondence: Huabing Zhao, zhaohuabing@tust.edu.cn

Table S1 KEGG pathway enriched in PPI network

Term ID	Term description	Observed gene count	Background gene count	Strength	Signal	False discovery rate
smu01100	Metabolic pathways	73	378	0.39	0.91	1.04E-11
smu02010	ABC transporters	24	90	0.53	0.73	4.62E-05
smu02024	Quorum sensing	18	51	0.65	0.81	4.62E-05
smu00190	Oxidative phosphorylation	8	9	1.05	0.88	0.00023
smu00230	Purine metabolism	12	34	0.65	0.61	0.0013
smu01212	Fatty acid metabolism	7	10	0.94	0.68	0.0016
smu01110	Biosynthesis of secondary metabolites	29	170	0.33	0.45	0.0021
smu00061	Fatty acid biosynthesis	7	12	0.87	0.61	0.0028
smu03010	Ribosome	13	53	0.49	0.44	0.0077
smu01501	beta-Lactam resistance	5	11	0.76	0.35	0.042

Table S2 Changes in the expression of genes implicated

Gene ID	Gene name	Function	Log2 FC	*p adj* value
SMU_1128	*ciaH*	putative histidine kinase sensor CiaH	-2.02	<0.01
SMU_1129	*ciaR*	putative response regulator CiaR	-1.74	<0.01
SMU_2164	*htrA*	serine protease HtrA	-1.69	<0.01
SMU_1954	*groEL*	putative chaperonin GroEL	-2.52	<0.01
SMU_1672	*clpP*	putative ATP-dependent Clp protease%2C proteolytic subunit	-1.35	<0.01
SMU_1396	*gbpC*	glucan-binding protein C%2C GbpC	-1.57	<0.01
SMU_82	*dnaK*	heat shock protein%2C DnaK (HSP-70)	-2.55	<0.01

Table S3 Quantitative CLSM analysis of *S. mutans* biofilms (24 h) after asiatic acid treatment

Treatment	Maximum Thickness (μm)	SYTO 9 RFI (Live cells)	PI RFI (Dead cells)	Alexa Fluor 647 RFI (EPS)
NC (saline)	84.94 ± 4.82	1.01 ± 0.11	1.01 ± 0.11	1.01 ± 0.10
31.25 μM AA	42.08 ± 3.73***	0.89 ± 0.10	0.78 ± 0.12**	0.95 ± 0.18
62.5 μM AA	31.58 ± 0.40***	0.78 ± 0.23**	0.61 ± 0.22***	0.64 ± 0.23***
125 μM AA	25.99 ± 0.42***	0.36 ± 0.07***	0.21 ± 0.06***	0.22 ± 0.11***
VC (1.25% DMSO)	76.80 ± 8.12	0.85 ± 0.10	0.83 ± 0.08*	0.97 ± 0.10
PC (0.12% CHX)	22.55 ± 2.42***	0.26 ± 0.04***	0.10 ± 0.02***	0.03 ± 0.01***

PC: positive control (0.12% CHX); NC: negative control (saline); VC: vehicle control (1.25% DMSO). **p* < 0.05 compared to NC. ***p* < 0.01 compared to NC. ****p* < 0.001 compared to NC.

Table S4 Quantitative CLSM analysis of *S. mutans* biofilms (48 h) after asiatic acid treatment

Treatment	Maximum Thickness (μm)	SYTO 9 RFI (Live cells)	PI RFI (Dead cells)	Alexa Fluor 647 RFI (EPS)
NC (saline)	146.77 ± 2.95	1.00 ± 0.17	1.00 ± 0.25	1.00 ± 0.27
31.25 μM AA	113.33 ± 4.16***	0.87 ± 0.38	0.98 ± 0.0.44	0.81 ± 0.40
62.5 μM AA	95.33 ± 1.15***	0.16 ± 0.02***	0.15 ± 0.02***	0.07 ± 0.02***
125 μM AA	54.11 ± 9.46***	0.15 ± 0.12***	0.16 ± 0.05***	0.05 ± 0.01***
VC (1.25% DMSO)	143.94 ± 5.39	0.94 ± 0.17	1.03 ± 0.17	1.13 ± 0.25
PC (0.12% CHX)	42.00 ± 5.29***	0.36 ± 0.05***	0.11 ± 0.05***	0.05 ± 0.01***

PC: positive control (0.12% CHX); NC: negative control (saline); VC: vehicle control (1.25% DMSO). *$p < 0.05$ compared to NC. **$p < 0.01$ compared to NC. ***$p < 0.001$ compared to NC.

Table S5 Primers

Primer name	Sequence（5'-3'）
16S-F	AGCGTTGTCCGGATTTATTG
16S-R	CTACGCATTTCACCGCTACA
ciaH-F	TCAGTTGCGGACAATGGTCA
ciaH-R	ACCCACCCTTTTGTCGTGTT
ciaR-F	TGTATGAAGCAGAGAGTGGCG
ciaR-R	CGTGTCCTTTGTCATCCAAACC
htrA-F	TGGTAATTCTGGTGGCGCTT
htrA-R	AGCCCATTCCTTCAACAGCA
groEL-F	CTGCTAACGGGGAATGGGTT
groEL-R	ATGCTTGGATCCATAGCGGG
clpP-F	CGGGTGGTTCTGTTTCAGCT
clpP-R	TCCTTTGGCACCGCTAGAAG
gbpC-F	GTCAATGCTGATGGAACGCC
gbpC-R	GTGTTGGTTTAGCTCCCGGA
dnaK-F	CTCCTGTTCGTCAAGCCCTT
dnaK-R	TTGAATGGCAGCCCCCATAG
